# CD8 T Cells Contribute to Vaccine Protection Against SARS-CoV-2 in Macaques

**DOI:** 10.1126/sciimmunol.abq7647

**Published:** 2022-08-09

**Authors:** Jinyan Liu, Jingyou Yu, Katherine McMahan, Catherine Jacob-Dolan, Xuan He, Victoria Giffin, Cindy Wu, Michaela Sciacca, Olivia Powers, Felix Nampanya, Jessica Miller, Michelle Lifton, David Hope, Kevin Hall, Nicole P. Hachmann, Benjamin Chung, Tochi Anioke, Wenjun Li, Jeanne Muench, Adrienne Gamblin, Mona Boursiquot, Anthony Cook, Mark G. Lewis, Hanne Andersen, Dan H. Barouch

**Affiliations:** ^1^ Center for Virology and Vaccine Research, Beth Israel Deaconess Medical Center, Boston, MA 02115, USA.; ^2^ Ragon Institute of MGH, MIT, and Harvard, Cambridge, MA, USA.; ^3^ University of Massachusetts, Lowell, MA 01854, USA.; ^4^ Bioqual, Rockville, MD 20852, USA.

## Abstract

Spike-specific neutralizing antibodies (NAbs) are generally considered key correlates of vaccine protection against SARS-CoV-2 infection. Recently, robust vaccine prevention of severe disease with SARS-CoV-2 variants that largely escape NAb responses has been reported, suggesting a role for other immune parameters for virologic control. However, direct data demonstrating a role of CD8^+^ T cells in vaccine protection has not yet been reported. In this study, we show that vaccine-elicited CD8^+^ T cells contribute substantially to virologic control following SARS-CoV-2 challenge in rhesus macaques. We vaccinated 30 macaques with a single immunization of the adenovirus vector-based vaccine Ad26.COV2.S or sham and then challenged them with 5x10^5^ TCID_50_ SARS-CoV-2 B.1.617.2 (Delta) by the intranasal and intratracheal routes. All vaccinated animals were infected by this high-dose challenge but showed rapid virologic control in nasal swabs and bronchoalveolar lavage by day 4 following challenge. However, administration of an anti-CD8α or anti-CD8β depleting monoclonal antibody in vaccinated animals prior to SARS-CoV-2 challenge resulted in higher levels of peak and day 4 virus in both the upper and lower respiratory tracts. These data demonstrate that CD8^+^ T cells contribute substantially to vaccine protection against SARS-CoV-2 replication in macaques.

## Teaser

In vivo CD8 T cell depletion in vaccinated macaques prior to SARS-CoV-2 challenge resulted in higher viral loads following challenge.

## INTRODUCTION

Antibody responses are generally considered key correlates of vaccine protection against SARS-CoV-2 infection (*1*). However, preclinical and clinical studies have suggested that CD8^+^ T cell responses may also contribute to natural immunity against SARS-CoV-2, particularly when antibody responses are subprotective (*2*–*4*). Moreover, cellular immune responses have shown greater durability (*5*–*8*) and cross-reactivity (*9*–*13*) than serum neutralizing antibody (NAb) responses against SARS-CoV-2 variants. In addition, recent studies that have reported that the mRNA vaccine BNT162b2 and the adenovirus vector-based vaccine Ad26.COV2.S provided 70% and 85% efficacy, respectively, against hospitalization with the SARS-CoV-2 Omicron BA.1 variant in South Africa (*14*, *15*), largely in the absence of Omicron-specific NAbs, suggesting the importance of other immune responses in protection against severe disease.

Virus-specific CD8^+^ T cells recognize and eliminate virally infected cells. However, a direct role of CD8^+^ T cell responses in vaccine protection against SARS-CoV-2 has not yet been established. Cellular immune responses were not included in the immune correlates analyses in any of the phase 3 human trials of SARS-CoV-2 vaccines completed to date. We therefore evaluated the contribution of CD8^+^ T cells to protective efficacy by the single-shot Ad26.COV2.S vaccine (*16*, *17*) against a high-dose challenge with the SARS-CoV-2 B.1.617.2 (Delta) variant in rhesus macaques.

## RESULTS

### Study design

**Fig. 1.**
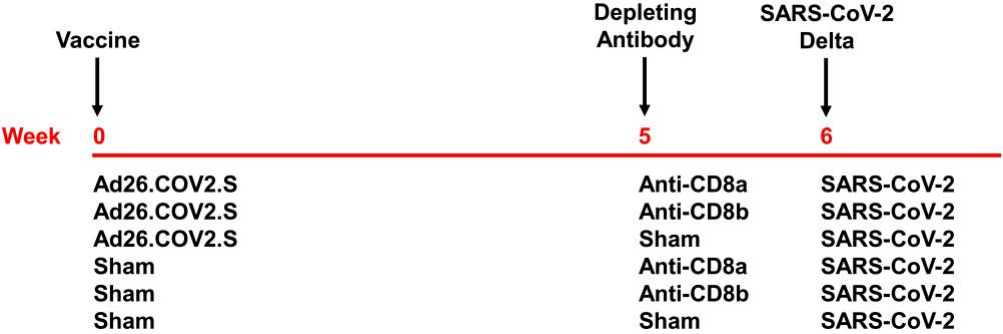
Study schema. Rhesus macaques were vaccinated with Ad26.COV2.S or sham at week 0 and then received anti-CD8α, anti-CD8β, or sham mAbs at week 5 (N = 6/group) prior to SARS-CoV-2 B.1.617.2 (Delta) challenge at week 6.

To evaluate the contribution of CD8^+^ T cell responses to vaccine protection against SARS-CoV-2, we immunized 30 rhesus macaques with 5x10^10^ viral particles of Ad26.COV2.S (Janssen / Johnson & Johnson; N = 15) or a sham injection (N = 15) by the intramuscular route at week 0. Animals received CD8-depleting mAbs at week 5 and then were challenged with SARS-CoV-2 B.1.617.2 (Delta) at week 6 (**Fig. 1**). In each vaccine arm (N = 5/group), we administered 50 mg/kg of the anti-CD8α CDR-grafted rhesus IgG1 antibody (MT807R1) or the anti-CD8β CDR-grafted rhesus IgG1 antibody (CD8b255R1) or an isotype-matched sham mAb by the intravenous route (**Fig. 1**). The anti-CD8α mAb results in more robust CD8 depletion than the anti-CD8β mAb, but the anti-CD8α mAb depletes both CD8^+^ T cells and NK cells, whereas the anti-CD8β mAb is specific for CD8^+^ T cells (*2*, *18*, *19*).

### Humoral and cellular immune responses to Ad26.COV2.S vaccine

**Fig. 2.**
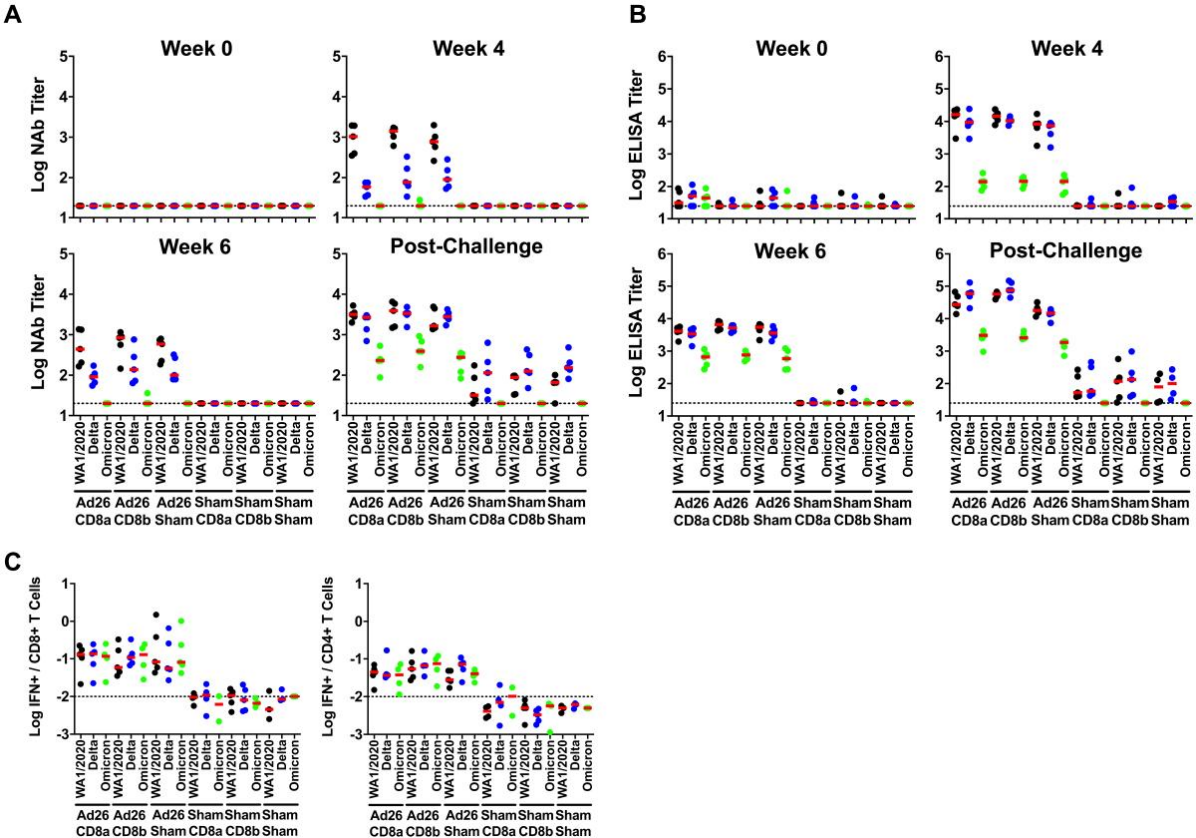
Immune responses following vaccination. Antibody responses at weeks 0, 4, and 6 following vaccination with Ad26.COV2.S and following challenge. **A,** Neutralizing antibody (NAb) titers by a luciferase-based pseudovirus neutralization assay. **B**, Receptor binding domain (RBD)-specific binding antibody titers by ELISA. **C**, Pooled peptide Spike-specific IFN-γ CD8^+^ and CD4^+^ T cell responses by intracellular cytokine staining assays at week 2 following vaccination with Ad26.COV2.S. Responses were measured against the SARS-CoV-2 WA1/2020 (black), B.1.617.2 (Delta; blue), and B.1.1.529 (Omicron; green) variants. Dotted lines represent limits of quantitation. Medians (red bars) are shown.

We evaluated vaccine-elicited neutralizing antibody responses by luciferase-based pseudovirus neutralization assays (*20*), receptor binding domain (RBD)-specific binding antibody responses by ELISA, and Spike (S)- and RBD-specific binding antibody responses by electrochemiluminescence assays (ECLA) (*21*). The three groups that received Ad26.COV2.S developed NAbs at week 4 against the vaccine-matched SARS-CoV-2 WA1/2020 strain, lower cross-reactive NAbs against the SARS-CoV-2 B.1.617.2 (Delta) variant, and minimal NAbs against the SARS-CoV-2 B.1.1.529 (Omicron BA.1) variant (**Fig. 2****A**). At week 6, median NAb titers in the vaccinated animals were 442–840 against WA1/2020, 90–137 against Delta, and < 20 against Omicron BA.1 after single-shot Ad26.COV2.S vaccination (**Fig. 2****A**). Similar trends were observed with RBD-specific ELISA titers (**Fig. 2****B**) and RBD- and S-specific ECLA titers (**Figs. S1, S2**), although binding antibody titers were generally more cross-reactive against SARS-CoV-2 variants than NAb titers. Antibody titers in this study were comparable with prior studies of Ad26.COV2.S (*17*, *22*–*25*).

We evaluated vaccine-elicited T cell responses by pooled peptide S-specific intracellular cytokine staining assays (*10*, *17*). The three groups that received Ad26.COV2.S developed IFN-γ CD8^+^ and CD4^+^ T cell responses at week 2 against the vaccine-matched SARS-CoV-2 WA1/2020 strain, with comparable responses against the Delta and Omicron BA.1 variants (**Fig. 2****C**), as expected (*10*). Median S-specific CD8^+^ T cell responses in the vaccinated animals were 0.054%–0.129% against WA1/2020, 0.056%–0.135% against Delta, and 0.081%–0.129% against Omicron BA.1 (**Fig. 2****C**).

We also assessed RBD-specific memory B cell responses by antigen-specific B cell staining assays (*23*, *25*). The three groups that received Ad26.COV2.S developed median RBD-specific memory B cell responses at week 2 that were 0.11% against the vaccine-matched SARS-CoV-2 WA1/2020 strain and 0.52% against the SARS-CoV-2 B.1.617.2 (Delta) variant (**Fig. S3**). Memory B cells exhibited primarily an activated memory phenotype (CD21^−^CD27^+^) (**Fig. S4**).

### Contribution of CD8^+^ T cells to protective efficacy of Ad26.COV2.S vaccine

At week 5, we infused 50 mg/kg of the depleting anti-CD8α CDR-grafted rhesus IgG1 antibody (MT807R1) or the depleting anti-CD8β CDR-grafted rhesus IgG1 antibody (CD8b255R1) or an isotype-matched sham mAb (N = 5/group) by the intravenous route. The anti-CD8α mAb led to profound depletion of CD8^+^CD3^+^ T cells to undetectable levels in peripheral blood (**Fig. S5**). Prior studies from our laboratory and others also show that this anti-CD8α mAb also effectively depletes CD8^+^ T cells in tissues (*2*, *18*, *19*, *26*). The anti-CD8β mAb led to less complete but still substantial depletion of CD8^+^CD3^+^ T cells to median levels of 51–57 cells per μl (**Fig. S5**), as expected (*2*, *19*).

**Fig. 3.**
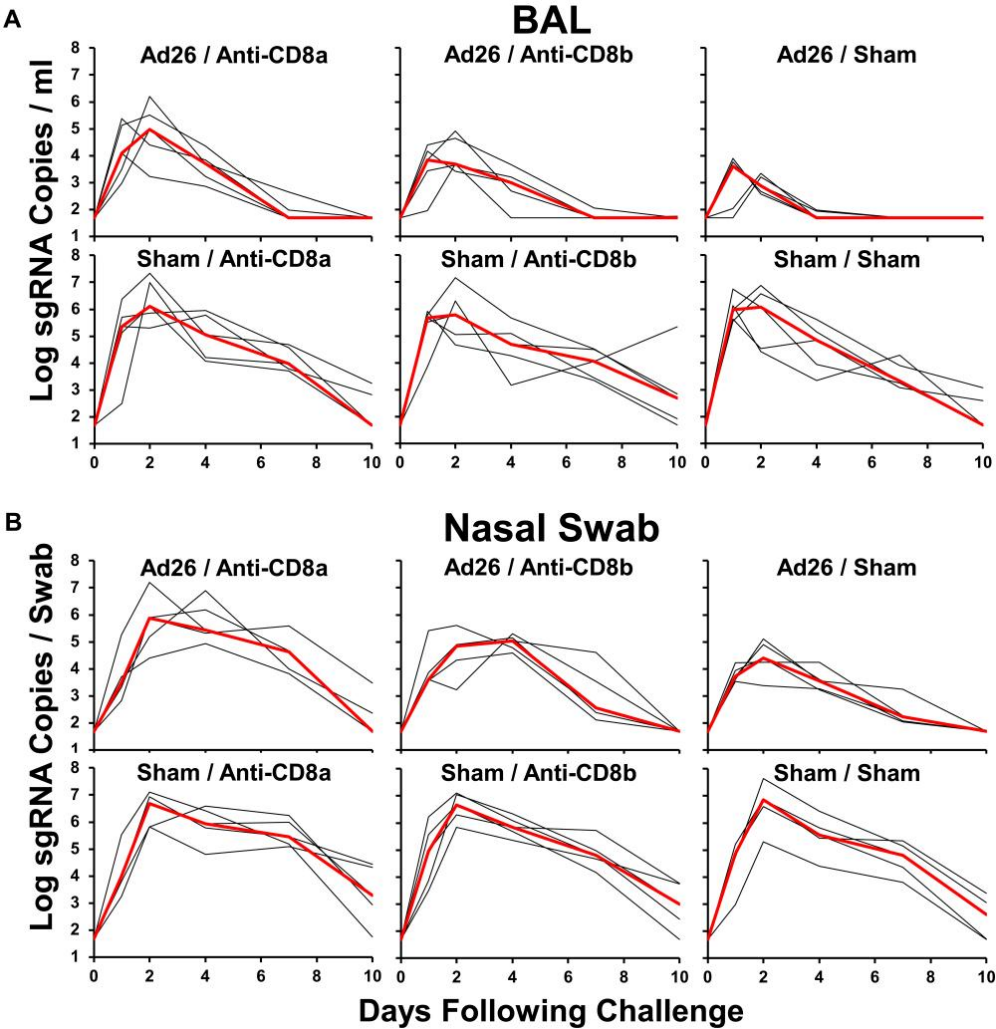
Viral loads following SARS-CoV-2 challenge. A, B, Log subgenomic RNA (sgRNA) copies/ml in bronchoalveolar lavage (BAL) following SARS-CoV-2 Delta challenge. Log subgenomic RNA (sgRNA) copies/swab in nasal swabs (NS) following SARS-CoV-2 Delta challenge. Medians (red lines) are shown.

At week 6, we challenged all animals with 5x10^5^ TCID50 SARS-CoV-2 B.1.617.2 (Delta) by the intranasal and intratracheal routes. Given the single-shot Ad26.COV2.S immunization, the borderline median NAb titers of 90–137 against Delta, and the high-dose Delta challenge, all animals were infected as expected. We assessed viral loads in bronchoalveolar lavage (BAL) (**Fig. 3****A**) and in nasal swabs (NS) (**Fig. 3****B**) on days 0, 1, 2, 4, 7, and 10 following challenge by E subgenomic RNA (sgRNA) RT-PCR (*27*).

**Fig. 4.**
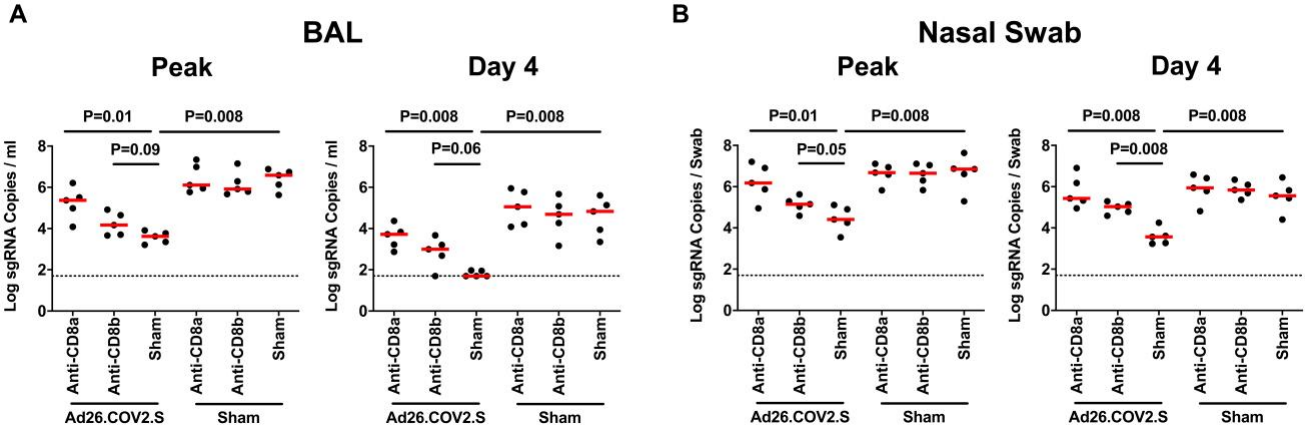
Comparison of peak and day 4 viral loads. A, B, Log subgenomic RNA (sgRNA) copies/ml in bronchoalveolar lavage (BAL) at peak and on day 4 following SARS-CoV-2 Delta challenge. Log subgenomic RNA (sgRNA) copies/swab in nasal swabs (NS) at peak and on day 4 following SARS-CoV-2 Delta challenge. Dotted lines represent limits of quantitation. Medians (red bars) are shown. P values reflect two-sided Mann-Whitney tests.

In sham controls, median peak log_10_ sgRNA levels in BAL were 5.92–6.58 RNA copies/ml and were not detectably impacted by CD8α or CD8β depletion. In vaccinated animals, median peak log_10_ sgRNA levels in BAL were 3.62 RNA copies/ml without CD8 depletion, 4.17 RNA copies/ml with CD8β depletion, and 5.37 RNA copies/ml with CD8α depletion (**Fig. 4****A**). Ad26.COV2.S vaccination thus led to a 2.96 log_10_ reduction in median peak sgRNA in BAL (P = 0.008, two-sided Mann-Whitney test). CD8α depletion led to higher peak viral loads in BAL in vaccinated animals (P = 0.01), and a trend was observed with CD8β depletion (P = 0.09). On day 4, median peak log_10_ sgRNA levels in BAL were 4.69–5.06 RNA copies/ml in controls and were < 1.70, 3.00, and 3.72 RNA copies/ml in vaccinated animals without CD8 depletion, with CD8β depletion, and with CD8α depletion, respectively (**Fig. 4****A**). Ad26.COV2.S vaccination thus led to a > 3.13 log_10_ reduction in median day 4 sgRNA in BAL (P = 0.008). CD8α depletion led to higher day 4 viral loads in BAL in vaccinated animals (P = 0.008), and a trend was observed with CD8β depletion (P = 0.06).

In NS, median peak log_10_ sgRNA levels in sham controls were 6.65–6.85 RNA copies/swab and were 4.41, 5.15, and 6.18 RNA copies/swab in vaccinated animals without CD8 depletion, with CD8β depletion, and with CD8α depletion, respectively (**Fig. 4****B**). Ad26.COV2.S vaccination thus led to a 2.44 log_10_ reduction in median peak sgRNA in NS (P = 0.008), and both CD8α and CD8β depletion led to higher peak viral loads in NS in vaccinated animals (P = 0.01 and P = 0.05, respectively). On day 4, median log_10_ sgRNA levels in NS were 5.56–5.94 RNA copies/swab in controls and were 3.56, 5.03, and 5.43 RNA copies/swab in vaccinated animals without CD8 depletion, with CD8β depletion, and with CD8α depletion, respectively (**Fig. 4****B**). Ad26.COV2.S vaccination thus led to a 2.00 log_10_ reduction in median day 4 sgRNA in NS (P = 0.008), and both CD8α and CD8β depletion led to higher peak viral loads in NS in vaccinated animals (P = 0.008 and P = 0.008, respectively). Infectious virus titers on day 2 by TCID_50_ assays showed similar results (**Fig. S6**).

## DISCUSSION

The role of virus-specific CD8^+^ T cell responses in vaccine protection against severe disease with SARS-CoV-2 has to date remained unclear, in part because cellular immune responses were not included in the correlates of protection analyses in the phase 3 clinical trials of SARS-CoV-2 vaccines (*1*). In this study, we show that Ad26.COV2.S induced CD8^+^ T cells contribute substantially to virologic control of a high-dose heterologous challenge with the SARS-CoV-2 Delta variant in rhesus macaques. Depletion of CD8^+^ T cells in vaccinated animals led to higher peak and day 4 viral loads in the upper and lower respiratory tract following challenge. The greater effect on viral loads with CD8α depletion compared with CD8β depletion was likely the result of more profound CD8 depletion with the anti-CD8α mAb or possibly a functional role for NK cells.

Our data are consistent with prior studies that have shown that the two-shot BNT162b2 and Ad26.COV2.S vaccines still provided robust protection against severe disease with the Omicron BA.1 variant in South Africa, largely in the absence of Omicron-specific NAbs (*14*, *15*). Our laboratory and others have also demonstrated that T cell responses, unlike NAb responses, are highly cross-reactive against multiple SARS-CoV-2 variants including Omicron BA.1 (*8*–*13*), providing the immunologic context for these clinical observations. In addition, we previously reported that CD8^+^ T cell responses contributed to protection by natural immunity against re-challenge with SARS-CoV-2 in rhesus macaques with suboptimal antibody responses (*2*).

Our data support and extend these prior observations by showing that Ad26.COV2.S induced CD8^+^ T cell responses also directly contribute to virologic control following SARS-CoV-2 challenge in a stringent, high-dose, heterologous challenge model in rhesus macaques. However, this model only evaluated virologic control in animals following challenge, and thus our conclusions about the role of CD8^+^ T cell responses likely do not apply to protection against acquisition of infection, which probably requires high titers of NAbs. We previously demonstrated that antibodies alone, if adoptively transferred at a high titer, can block acquisition of SARS-CoV-2 infection in macaques (*2*). However, current clinical SARS-CoV-2 vaccines have only modest and transient efficacy in blocking acquisition of infection with the SARS-CoV-2 Omicron variant, even with third and fourth BNT162b2 boosts (*28*–*31*).

In conclusion, we show that CD8^+^ T cell responses contribute substantially to Ad26.COV2.S vaccine protection against SARS-CoV-2 replication in rhesus macaques. We speculate that CD8^+^ T cell responses may also contribute to virologic control following mRNA vaccination, although this has not yet been demonstrated. Both humoral and cellular immune responses are likely important for vaccine protection against SARS-CoV-2 severe disease. It is likely that CD8^+^ T cell responses contribute more to protection against SARS-CoV-2 variants that partially evade NAb responses such as Delta and Omicron, as compared to protection against the original SARS-CoV-2 Wuhan or WA1/2020 strains. Future studies should evaluate whether CD8^+^ T cell responses also contribute to SARS-CoV-2 vaccine protection in humans. Consistent with this perspective, approximately 70 investigators signed a letter to the FDA on April 21, 2022 encouraging the inclusion of T cell responses in addition to antibody titers for the evaluation of SARS-CoV-2 vaccines in humans, thus further raising awareness of the potential importance of cellular immunity for long-term vaccine protection, particularly against severe disease with SARS-CoV-2 variants (*32*).

## MATERIALS AND METHODS

### Animals and study design

30 outbred adult male and female rhesus macaques ages 4–6 years old were randomly allocated to 6 experimental groups (N = 5/group). All animals were singly housed at Bioqual, Inc. (Rockville, MD). Groups of animals were immunized by the intramuscular route in the quadriceps muscle with a single immunization at week 0 of 5x10^10^ viral particles of Ad26.COV2.S, which is equivalent to the human dose of this vaccine. Sham animals received an injection of saline. At week 5, animals (N = 5/group) received 50 mg/kg of the anti-CD8α CDR-grafted rhesus IgG1 antibody (MT807R1) or the anti-CD8β CDR-grafted rhesus IgG1 antibody (CD8b255R1) (MassBiologics, Mattapan, MA) or an isotype-matched sham mAb (prepared in the Barouch laboratory) by the intravenous route. At week 6, all animals were challenged with 5x10^5^ TCID_50_ SARS-CoV-2 B.1.617.2 (Delta) (BEI Resources SARS-Related Coronavirus 2, Isolate hCoV-19/USA/MDHP05647/2021, NR-55674, contributed by Dr. Andrew S. Pekosz) by the intranasal and intratracheal routes in a total volume of 2 mls. Following challenge, viral loads were assessed in bronchoalveolar lavage (BAL) and nasal swab (NS) samples by RT-PCR for E subgenomic RNA (sgRNA). Immunologic and virologic assays were performed blinded. All animal studies were conducted in compliance with all relevant local, state, and federal regulations and were approved by the Bioqual Institutional Animal Care and Use Committee (IACUC).

### Pseudovirus neutralizing antibody assay

The SARS-CoV-2 pseudoviruses expressing a luciferase reporter gene were used to measure pseudovirus neutralizing antibodies (*20*). In brief, the packaging construct psPAX2 (AIDS Resource and Reagent Program), luciferase reporter plasmid pLenti-CMV Puro-Luc (Addgene) and spike protein expressing pcDNA3.1-SARS-CoV-2 SΔCT were co-transfected into HEK293T cells (ATCC CRL_3216) with lipofectamine 2000 (ThermoFisher Scientific). Pseudoviruses of SARS-CoV-2 variants were generated by using WA1/2020 strain (Wuhan/WIV04/2019, GISAID accession ID: EPI_ISL_402124), B.1.617.2 (Delta, GISAID accession ID: EPI_ISL_2020950), or B.1.1.529 (Omicron BA.1, GISAID ID: EPI_ISL_7358094.2). The supernatants containing the pseudotype viruses were collected 48 h after transfection; pseudotype viruses were purified by filtration with 0.45-μm filter. To determine the neutralization activity of human serum, HEK293T-hACE2 cells were seeded in 96-well tissue culture plates at a density of 2.0 × 10^4^ cells per well overnight. Three-fold serial dilutions of heat-inactivated serum samples were prepared and mixed with 50 μl of pseudovirus. The mixture was incubated at 37 °C for 1 h before adding to HEK293T-hACE2 cells. After 48 h, cells were lysed in Steady-Glo Luciferase Assay (Promega) according to the manufacturer’s instructions. SARS-CoV-2 neutralization titers were defined as the sample dilution at which a 50% reduction (NT50) in relative light units was observed relative to the average of the virus control wells.

### Enzyme-linked immunosorbent assay (ELISA)

SARS-CoV-2 spike receptor-binding domain (RBD)-specific binding antibodies in serum were assessed by ELISA. 96-well plates were coated with 1 μg/mL of similarly produced SARS-CoV-2 WA1/2020, B.1.617.2 (Delta), or B.1.1.529 (Omicron BA.1) RBD protein in 1× Dulbecco phosphate-buffered saline (DPBS) and incubated at 4 °C overnight. Assay performance was similar for these four RBD proteins. After incubation, plates were washed once with wash buffer (0.05% Tween 20 in 1× DPBS) and blocked with 350 μL of casein block solution per well for 2 to 3 hours at room temperature. Following incubation, block solution was discarded and plates were blotted dry. Serial dilutions of heat-inactivated serum diluted in casein block were added to wells, and plates were incubated for 1 hour at room temperature, prior to 3 more washes and a 1-hour incubation with a 1 μg/mL dilution of anti–macaque IgG horseradish peroxidase (HRP) (Nonhuman Primate Reagent Resource) at room temperature in the dark. Plates were washed 3 times, and 100 μL of SeraCare KPL TMB SureBlue Start solution was added to each well; plate development was halted by adding 100 μL of SeraCare KPL TMB Stop solution per well. The absorbance at 450 nm was recorded with a VersaMax microplate reader (Molecular Devices). For each sample, the ELISA end point titer was calculated using a 4-parameter logistic curve fit to calculate the reciprocal serum dilution that yields an absorbance value of 0.2. Interpolated end point titers were reported.

### Electrochemiluminescence assay (ECLA)

ECLA plates (Meso Scale Discovery SARS-CoV-2 IgG, Panels 22, 23) were designed and produced for multiplex binding assays with up to 10 antigen spots in each well, including either Spike or RBD proteins from multiple SARS-CoV-2 variants (*21*). The plates were blocked with 50 μL of Blocker A (1% BSA in distilled water) solution for at least 30 minutes at room temperature shaking at 700 rpm with a digital microplate shaker. During blocking the serum was diluted to 1:5,000 or 1:50,000 in Diluent 100. The calibrator curve was prepared by diluting the calibrator mixture from MSD 1:10 in Diluent 100 and then preparing a 7-step 4-fold dilution series plus a blank containing only Diluent 100. The plates were then washed 3 times with 150 μL of Wash Buffer (0.5% Tween in 1x PBS), blotted dry, and 50 μL of the diluted samples and calibration curve were added in duplicate to the plates and set to shake at 700 rpm at room temperature for at least 2 h. The plates were again washed 3 times and 50 μL of SULFO-Tagged anti-Human IgG detection antibody diluted to 1x in Diluent 100 was added to each well and incubated shaking at 700 rpm at room temperature for at least 1 h. Plates were then washed 3 times and 150 μL of MSD GOLD Read Buffer B was added to each well and the plates were read immediately after on a MESO QuickPlex SQ 120 machine. MSD titers for each sample was reported as Relative Light Units (RLU) which were calculated as Sample RLU minus Blank RLU and then fit using a logarithmic fit to the standard curve. The upper limit of detection was defined as 2x10^6^ RLU for each assay and the signal for samples which exceeded this value at 1:5,000 serum dilution was run again at 1:50,000 and the fitted RLU was multiplied by 10 before reporting. The lower limit of detection was defined as 1 RLU and an RLU value of 100 was defined to be positive for each assay.

### Intracellular cytokine staining (ICS) assay

CD4^+^ and CD8^+^ T cell responses were quantitated by pooled peptide-stimulated intracellular cytokine staining (ICS) assays. Peptide pools were 16 amino acid peptides overlapping by 11 amino acids spanning the SARS-CoV-2 WA1/2020, B.1.617.2 (Delta), or B.1.1.529 (Omicron BA.1) Spike proteins (21st Century Biochemicals). 10^6^ peripheral blood mononuclear cells well were re-suspended in 100 μL of R10 media supplemented with CD49d monoclonal antibody (1 μg/mL; clone 9F10; BD Biosciences) and CD28 monoclonal antibody (1 μg/mL; clone CD28.2; BD Biosciences). Each sample was assessed with mock (100 μL of R10 plus 0.5% DMSO; background control), peptides (2 μg/mL), and/or 10 pg/mL phorbol myristate acetate (PMA) and 1 μg/mL ionomycin (Sigma-Aldrich) (100 μL; positive control) and incubated at 37 °C for 1 h. After incubation, 0.25 μL of GolgiStop and 0.25 μL of GolgiPlug in 50 μL of R10 was added to each well and incubated at 37 °C for 8 h and then held at 4 °C overnight. The next day, the cells were washed twice with DPBS, stained with aqua live/dead dye for 10 mins and then stained with predetermined titers of monoclonal antibodies against CD279 (clone EH12.1, BB700), CD38 (clone OKT10, PE), CD28 (clone 28.2, PE Cy5), CD4 (clone L200, BV510), CD95 (clone DX2, BUV737), CD8 (clone SK1, BUV805) for 30 min. Cells were then washed twice with 2% FBS/DPBS buffer and incubated for 15 min with 200 μL of BD CytoFix/CytoPerm Fixation/Permeabilization solution. Cells were washed twice with 1X Perm Wash buffer (BD Perm/Wash™ Buffer 10X in the CytoFix/CytoPerm Fixation/ Permeabilization kit diluted with Milli-Q water and passed through 0.22 μm filter) and stained with intracellularly with monoclonal antibodies (BD Biosciences) against Ki67 (clone B56, FITC), CD69 (clone TP1.55.3, ECD), IL-10 (clone JES3-9D7, PE CY7), IL-13 (clone JES10-5A2, BV421), TNF-α (clone Mab11, BV650), IL-4 (clone MP4-25D2, BV711), IFN-γ (clone B27; BUV395), CD45 (clone D058–1283, BUV615), IL-2 (clone MQ1-17H12, APC), CD3 (clone SP34.2, Alexa 700) for 30 min. Cells were washed twice with 1X Perm Wash buffer and fixed with 250 μL of freshly prepared 1.5% formaldehyde. Fixed cells were transferred to 96-well round bottom plate and analyzed by BD FACSymphony™ system. Data were analyzed using FlowJo v9.9.

### B cell immunophenotyping

PBMCs or inguinal LN cells were stained with Aqua live/dead dye for 20 minutes, washed with 2% FBS/DPBS buffer, and cells were suspended in 2% FBS/DPBS buffer with Fc Block (BD Biosciences) for 10 minutes (*23*). After blocking, samples were stained with monoclonal antibodies (BD Biosciences) against CD45 (clone D058–1283, brilliant ultraviolet (BUV) 805), CD3 (clone SP34.2, allophycocyanin (APC)-Cy7), CD7 (clone M-T701, Alexa Fluor700), CD123 (clone 6H6, Alexa Fluor 700), CD11c (clone 3.9, Alexa Fluor 700), CD19 (clone J3–119, phycoerythrin (PE)), CD20 (clone 2H7, PE-Cy5), IgD (IA6–2, PE), IgG (clone G18–145, BUV737), IgM (clone G20–127, BUV395), CD80 (clone L307.4, brilliant violet (BV) 786), CD95 (clone DX2, BV711), CD27 (clone M-T271, BUV563), CD21 (clone B-ly4, BV605), CD14 (clone M5E2, BV570). Samples were also stained with SARS-CoV-2 antigens, including biotinylated SARS-CoV-2 (WA1/2020) RBD proteins (Sino Biological), SARS-CoV-2 WA1/2020 RBD proteins (Sino Biological) labeled with fluorescein isothiocyanate (FITC), SARS-CoV-2 B.1.617.2 (Delta) RBD proteins (Sino Biological) labeled with APC and DyLight 405. Staining was done at 4 °C for 30 minutes. After staining, cells were washed twice with 2% FBS/DPBS buffer, followed by incubation with BV650 streptavidin (BD Pharmingen) for 10 minutes, then washed twice with 2% FBS/DPBS buffer. Cells were washed and fixed by 2% paraformaldehyde. All data were acquired on a BD FACSymphony flow cytometer. Subsequent analyses were performed using FlowJo software (BD Bioscience, v.9.9.6). For analyses, in singlet gate, dead cells were excluded by Aqua dye and CD45 was used as a positive inclusion gate for all leukocytes. Within class-switched memory B cell populations, gated as CD20^+^IgG^+^CD27^+^IgM^−^CD3^−^CD14^−^CD11c^−^CD123^−^CD7^−^, SARS-CoV-2 WA1/2020 RBD-specific B cells were identified as double positive for SARS-CoV-2 (WA1/2020) RBD labeled with different fluorescent probes, and SARS-CoV-2 (B.1.617.2) RBD-specific B cells were identified as double positive for SARS-CoV-2 (B.1.617.2) RBD proteins labeled with different fluorescent probes. SARS-CoV-2-specific B cells were phenotyped as activated memory B cells (CD21^−^CD27^+^) and resting memory B cells (CD21^+^CD27^+^).

### Subgenomic RT-PCR assay

SARS-CoV-2 E gene subgenomic RNA (sgRNA) was assessed by RT-PCR using primers and probes as previously described (*20*). A standard was generated by first synthesizing a gene fragment of the subgenomic E gene. The gene fragment was subsequently cloned into a pcDNA3.1(+) expression plasmid using restriction site cloning (Integrated DNA Technologies). The insert was in vitro transcribed to RNA using the AmpliCap-Max T7 High Yield Message Maker Kit (CellScript). Log dilutions of the standard were prepared for RT-PCR assays ranging from 1x10^10^ copies to 1x10^−1^ copies. Viral loads were quantified from bronchoalveolar lavage (BAL) fluid and nasal swabs (NS). RNA extraction was performed on a QIAcube HT using the IndiSpin QIAcube HT Pathogen Kit according to manufacturer’s specifications (Qiagen). The standard dilutions and extracted RNA samples were reverse transcribed using SuperScript VILO Master Mix (Invitrogen) following the cycling conditions described by the manufacturer. A Taqman custom gene expression assay (Thermo Fisher Scientific) was designed using the sequences targeting the E gene sgRNA. The sequences for the custom assay were as follows, forward primer, sgLeadCoV2.Fwd: CGATCTCTTGTAGATCTGTTCTC, E_Sarbeco_R: ATATTGCAGCAGTACGCACACA, E_Sarbeco_P1 (probe): VIC-ACACTAGCCATCCTTACTGCGCTTCG-MGBNFQ. Reactions were carried out in duplicate for samples and standards on the QuantStudio 6 and 7 Flex Real-Time PCR Systems (Applied Biosystems) with the thermal cycling conditions: initial denaturation at 95 °C for 20 seconds, then 45 cycles of 95 °C for 1 second and 60 °C for 20 seconds. Standard curves were used to calculate subgenomic RNA copies per ml or per swab. The quantitative assay sensitivity was determined as 50 copies per ml or per swab.

### TCID_50_ assay

Vero-TMPRSS2 cells (obtained from A. Creanga) were plated at 25,000 cells per well in DMEM with 10% FBS and gentamicin, and the cultures were incubated at 37 °C, 5.0% CO2. Medium was aspirated and replaced with 180 μl of DMEM with 2% FBS and gentamicin. Serial dilution of samples as well as positive (virus stock of known infectious titer) and negative (medium only) controls were included in each assay. The plates are incubated at 37 °C, 5.0% CO2 for 4 days. Cell monolayers were visually inspected for cytopathic effect. The TCID_50_ was calculated using the Read–Muench formula.

### Statistical analyses

Descriptive statistics were performed using GraphPad Prism 8.4.3, (GraphPad Software, San Diego, California). Virologic data were generated in duplicate and were compared by two-sided Mann-Whitney tests. The hypothesis regarding differences between CD8 depleted and non-depleted groups was pre-determined. P values less than 0.05 were considered significant.

## Supplementary Materials

### Other Supplementary Material for this manuscript includes the following:

Data file S1

MDAR Reproducibility Checklist

## Notes

View/request a protocol for this paper from *Bio-protocol*.
