# Supplementary material for: CD8 T Cells Contribute to Vaccine Protection Against SARS-CoV-2 in Macaques
Source: Sci Immunol. 2022 Aug 9:eabq7647. doi: 10.1126/sciimmunol.abq7647 (PMC9407944; doi:10.1126/sciimmunol.abq7647)
Supplement: Supplementary file 1 — Figs. S1 to S6 [file sciimmunol.abq7647_sm.pdf]

Science Immunology
AAAS

Supplementary Materials for

CD8 T cells contribute to vaccine protection against SARS-CoV-2 in macaques

Jinyan Liu *et al.*

Corresponding author: Dan H. Barouch, dbarouch@bidmc.harvard.edu

Sci. Immunol. **7**, eabq7647 (2022)
DOI: 10.1126/sciimmunol.abq7647

The PDF file includes:

Figs. S1 to S6

Other Supplementary Material for this manuscript includes the following:

Data file S1
MDAR Reproducibility Checklist

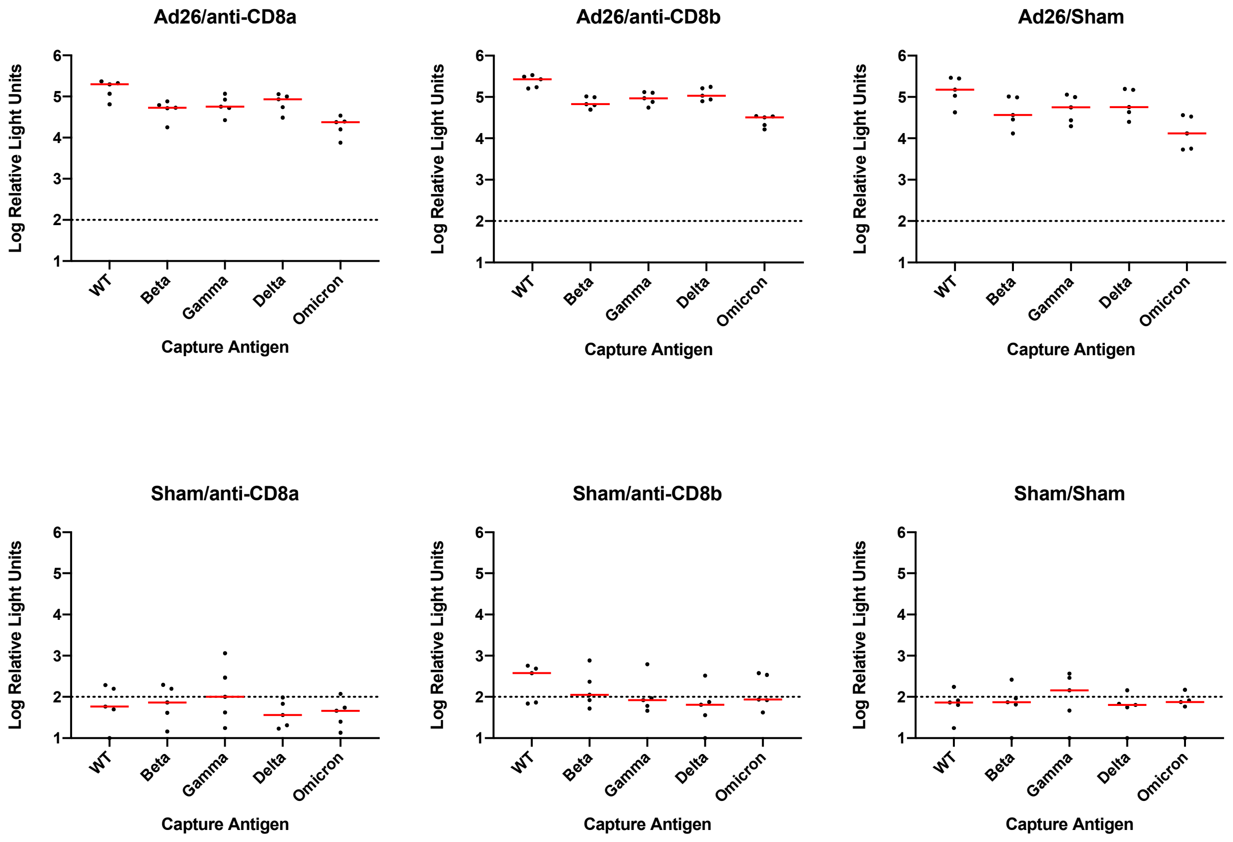

Figure S1. Spike-specific binding antibody responses following vaccination. Spike-specific antibody responses against multiple variants are shown at week 4 following vaccination with the Meso Scale Discovery electrochemiluminescence assay (ECLA). Dotted lines represent limits of quantitation. Medians (red bars) are shown.

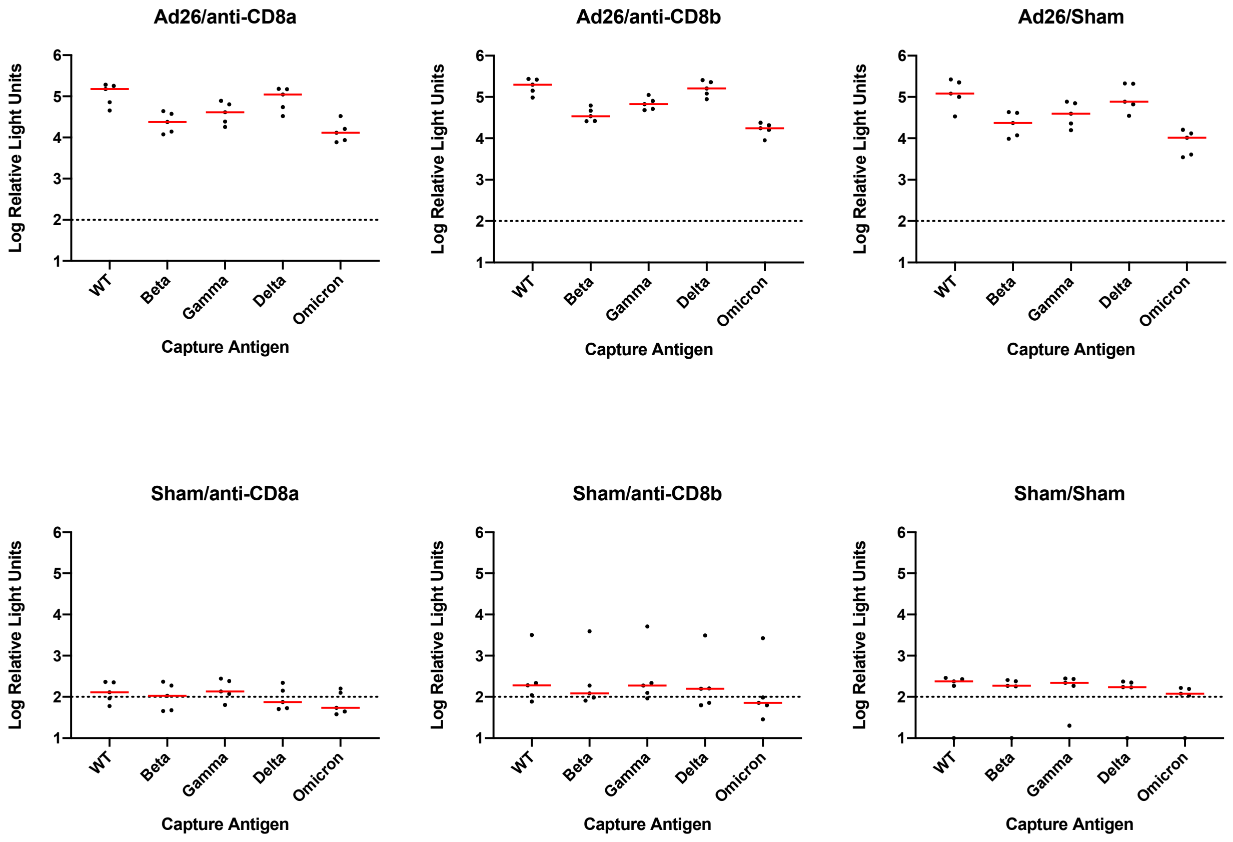

Figure S2. RBD-specific binding antibody responses following vaccination. RBD-specific antibody responses against multiple variants are shown at week 4 following vaccination with the Meso-Scale Discovery electrochemiluminescence assay (ECLA). Dotted lines represent limits of quantitation. Medians (red bars) are shown.

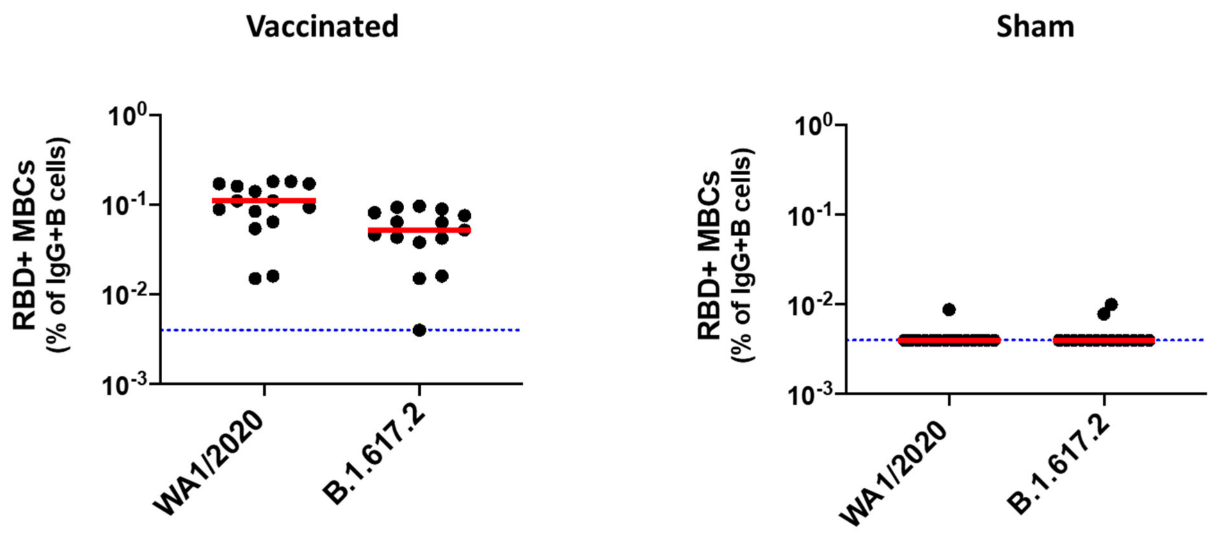

Figure S3. RBD-specific memory B cell responses following vaccination. WA1/2020 and B.1.617.2 (Delta) RBD-specific memory B cell (MBC) responses in peripheral blood at week 4 following vaccination. Dotted lines represent limits of quantitation. Medians (red bars) are shown.

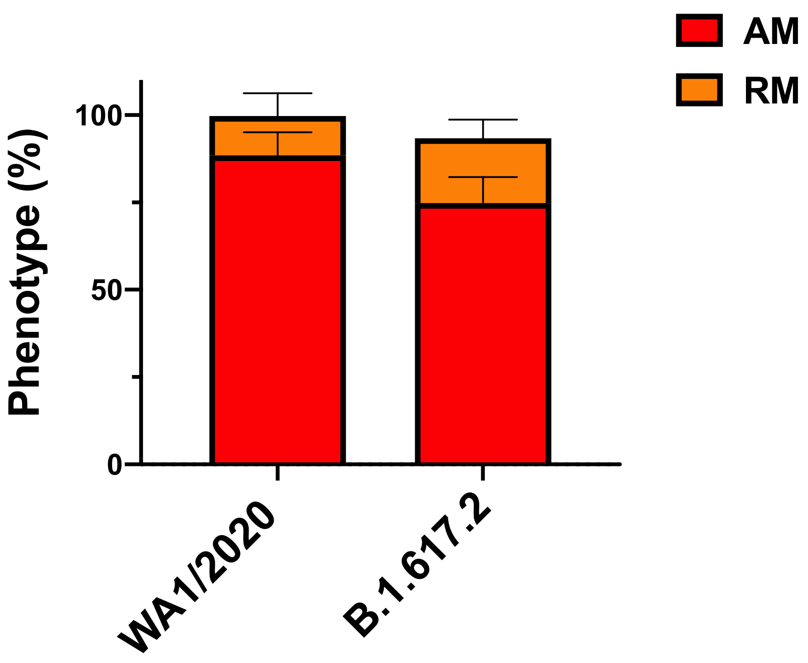

Figure S4. Phenotype of RBD-specific memory B cells following vaccination. Phenotype of WA1/2020 and B.1.617.2 (Delta) RBD-specific memory B cell (MBC) responses in peripheral blood at week 4 following vaccination. Red, activated memory (AM) B cells. Orange, resting memory (RM) B cells.

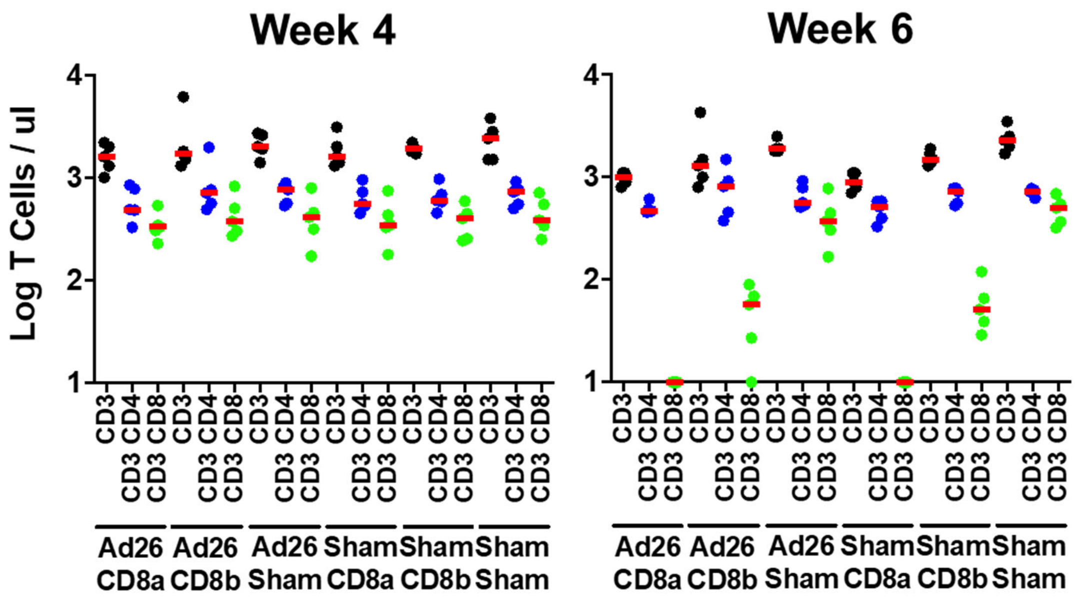

Figure S5. CD8 depletion. $CD3^+$, $CD8^+CD3^+$, and $CD4^+CD3^+$ cells in peripheral blood at week 4 prior to CD8 depletion and at week 6 after CD8 depletion. At week 5, we administered 50 mg/kg of the anti-CD8α CDR-grafted rhesus IgG1 antibody (MT807R1) or the anti-CD8β CDR-grafted rhesus IgG1 antibody (CD8b255R1) or an isotype-matched sham mAb (N=5/group) by the intravenous route in the Ad26.COV2.S and the sham vaccinated animals.

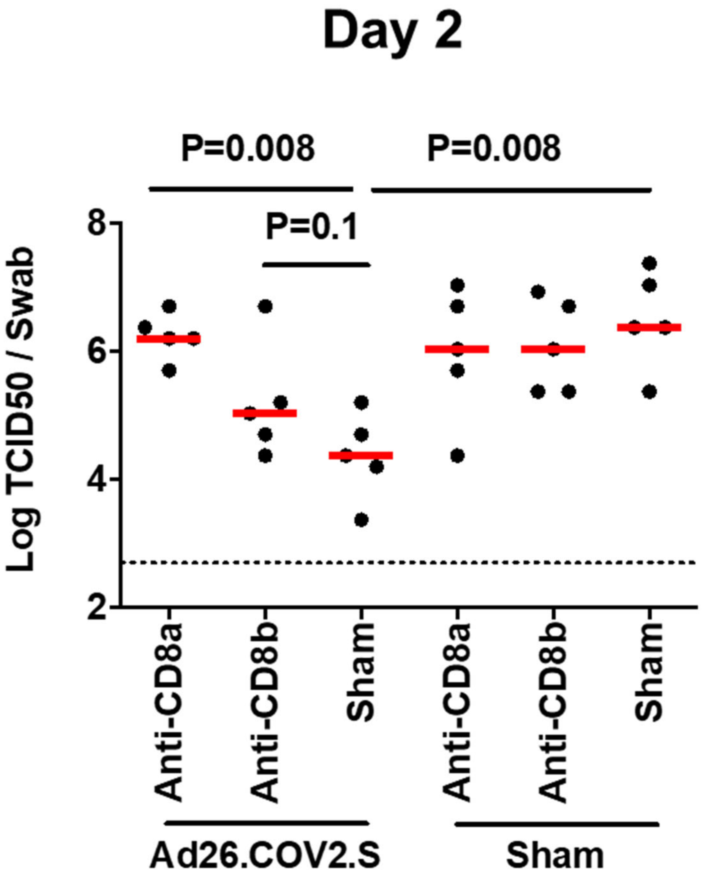

Figure S6. $TCID_{50}$ titers following SARS-CoV-2 challenge. Log $TCID_{50}$ copies/swab in nasal swabs (NS) on day 2 following SARS-CoV-2 Delta challenge. Medians (red lines) are shown.
